# Remarks on the Bearing of Some Modern Doctrines of Pathology and Animal Chemistry on the Treatment of Tubercular Consumption

**Published:** 1848

**Authors:** E. J. Marsh

**Affiliations:** Paterson


### ARTICLE XV.

*Remarks on the Bearing of some Modern Doctrines of Pathology and Animal Chemistry on the Treatment of Tubercular Consumption.* By E. J. Marsh, M. D.

Tubercular Consumption has always claimed and received a large share of the time and attention of the profession; the wide extent of its ravages, the insidious nature of its approach, the character of its victims, the usual fatality of its attack, and the confessed inability of medicinal agents even to stay its progress, have all contributed to invest this malady with a high degree of interest both for the profession and lay-public; and every attempt to throw light upon its nature and treatment, has been received with kindness and attention.

Some recent investigations and discoveries of European pathologists and chemists appear to me to have a bearing upon this subject, and I have thought that a brief statement of these doctrines and facts with inferences drawn from them, may not be without interest and profit.

Bennet, and other morbid anatomists have stated as the result of numerous dissections, that cicatrices of ulcers of the lungs were found more frequently in the bodies of spirit-drinkers dying of other diseases than phthisis, than in persons of different habits. These cicatrices were proofs of the existence of former cavities which had become healed up; and they were met with, and that not rarely, for the most part in persons who had been spirit-drinkers, proving conclusively that ulcers of the lungs may become healed.

It is well known that the blood is more highly arterialized and abounds more in fibrine in phthisis pulmonalis than in most other disorders of the economy; and this condition of the blood continues through the whole course of the disease, when it proceeds to a fatal termination. Rokinstansky, one of the most profound and distinguished of the German pathologists, states that tuberculosis depends upon a fibrinous trasis of the blood, and that all attempts at staying the procress of the disease will be vain and futile, unless this condigon of the blood be changed: and that if this crisis of the tilood be changed the disease will be checked, and in many eases the ulcers will heal, It has long been known to practibal physicians, that certain conditions of the system suspendcd the progress of consumption, as pregnancy; and that cereain diseases such as chronic bronchial affections, and some diseases of the heart prevented or stayed the pulmonary affection. The cause of this has not been well understood, and has received different explanations. Rokinstansky states that these conditions and diseases present mechanical obstacles to the transmission of the blood through the lungs, and prevent its arterialization, keeping it in a venous condition. This venosity of the blood prevents the formation of that fibrinous crasis, on which the developement and deposit of tuberculous matter depends.

Intermittent fever also prevents the developement of tuberculosis, probably by some action on the blood, as this poison appears to exert a specific effect on the liver and spleen, organs particularly connected with the venous circulation.

To prevent or cure tuberculosis, it should be our endeavor to change the fibrinous condition of the vital fluid; and causes which produce and maintain a venosity of the blood, will effect this.

It has been proved by the experiments and facts of Brodie, Paris, and others, that alcoholic drinks taken into the stomach, pass undecomposed by absorption, or endosmose, into the blood vessels, and circulates in a free state of the blood.

Leibig, our highest authority in animal chemistry, states that alcohol circulating in the blood unites with the oxygen in that fluid, and forms with it carbonic acid, keeping it in a venous state, and preventing that fibrinous crasis which is the origin of tuberculosis; carbonic acid, it is well known, is the element which causes the venosity of the blood. Such are the results of the investigations of different and independent laborers in the vineyard of truth. Bennet finds that tuberculous cavities are found more frequently healed in the lungs of spirit-drinkers than of any other class; Rokinstansky shows that an altered condition of the blood is necessary for the cure of tuberculosis, and that this altered condition is a state of venosity; and Leibig teaches that the alcohol which spirit-drinkers take into the stomach passes into the blood vessels, and there uniting with oxygen forms carbonic acid, and produces a venous condition of that fluid.

Without wishing to give any countenance to intemperance, may I not ask the profession, whether in view of these statements, the total prohibition of spirituous drinks to all persons, especially to those pre-disposed to tubercles, is not likely to be attended with ill effects?

Whether the moderate use of alcoholic drinks ought not to be recommended to persons disposed to consumption, and the more free use of them be recommended to persons laboring under the disease?

Whether consumption of the lungs be not more prevalent than formerly, and whether the disease be not increasing in those communities, and among those persons who most strictly abstain from all spirituous beverages?

Whether the fibrinous condition of the blood can be altered by any system of diet?

Paterson, June, 1848. [*New Jersey Med. Reporter.*

